# Neutrophil–lymphocyte ratio as a prognostic factor for minute clear cell renal cell carcinoma diagnosed using multi-slice spiral CT

**DOI:** 10.1097/MD.0000000000026292

**Published:** 2021-06-11

**Authors:** Li Chen, Lingjun Qi, Jing Zhang, Qian Ma, Xiaoxin Chai

**Affiliations:** Department of Radiology, Urumqi Friendship Hospital, No. 558 Shengli Road, Tianshan District, 830049 Urumqi, P.R. China.

**Keywords:** minute clear cell renal cell carcinoma, multi-slice spiral CT, neutrophil–lymphocyte ratio, overall survival, prognosis, regression

## Abstract

Minute clear cell renal cell carcinoma (MccRCC) has a diameter of <1.5 cm and can be diagnosed using multi-slice spiral CT (MSCT). Recently, the role of the neutrophil–lymphocyte ratio (NLR) in the development of MccRCC has attracted attention. This study aimed to further explore the relationship between the NLR and MccRCC.

This was a prospective study of 100 patients who were diagnosed with MccRCC using MSCT at Urumqi Friendship Hospital, China. The study investigated a series of pretreatment factors, including NLR and patients’ general clinical data. Statistical methods employed included Pearson's chi-square test, Spearman-rho correlation test, Cox regression analysis, and receiver operator characteristic curve analysis.

Based on Pearson's χ^2^, Spearman-rho test, and univariate/multivariate Cox regression analysis, the overall survival of patients with MccRCC was shown to be significantly related to NLR (*P* < .001). NLR (hazard ratio = 50.676, 95%CI, 17.543–146.390, *P* < .001) is a significant independent risk-factor for MccRCC. A receiver operator characteristic curve was plotted to examine specificity and sensitivity between NLR and MccRCC (area under curve = 0.958, *P* < .001).

The level of the NLR plays a crucial role in the survival of patients with MccRCC, as diagnosed with MSCT. The higher the NLR, the worse the prognosis for patients with MccRCC.

## 1 Introduction

Clear cell renal cell carcinoma originates from renal tubular epithelial cells, and its incidence is second only to bladder cancer among urinary tract carcinomas. It accounts for 85% to 90% of primary malignant renal tumors, with a mortality rate as high as 40%.^[1]^ Therefore, this is a highly malignant tumor of the urinary system.^[2]^ The etiology of clear cell renal cell carcinoma is unknown and may be related to obesity, smoking, long-term hemodialysis, or occupational factors. Clear cell renal cell carcinoma is the most common pathological type of renal carcinoma that is not sensitive to conventional radiotherapy or chemotherapy.^[3]^ As most patients with clear cell renal cell carcinoma have atypical clinical symptoms, currently the disease is mainly detected through the use of medical imaging examination. Almost 50% of renal cancers are discovered by chance, and about 25% of patients have metastases at the time of diagnosis.^[4,5]^

Small renal cell carcinoma (SRCC) refers to renal cell carcinomas with a diameter of <2.5 cm, while minute clear cell renal cell carcinoma (MccRCC) usually has a diameter of <1.5 cm.^[6,7]^ MccRCC has the characteristics of being clinically asymptomatic, with small lesions, and it is easy to miss during an investigation. It has been confirmed that tumor diameter is related to prognosis in renal cell carcinoma. In view of these characteristics of MccRCC, clinicians have found that early detection can be achieved through imaging examinations, such as multi-slice computed tomography (MSCT) and magnetic resonance imaging (MRI), leading to a better prognosis.

MSCT is developed on the basis of single-layer spiral CT, which can obtain better three-dimensional (3D) reconstructed images, with larger scanning coverage, shorter scanning time, and higher z-axis resolution.^[8]^ It has been found that the application of multilayer helixes in clinical practice can greatly improve the detection rate and differential diagnosis of kidney tumors, especially microtumors,^[9]^ and is more conducive to the early diagnosis of the disease.^[10]^ MSCT can help to avoid small lesions being missed, which is beneficial to the detection and characterization of metastatic renal cell carcinoma.

The neutrophil–lymphocyte ratio (NLR) is the ratio of the neutrophil to lymphocyte count.^[11]^ In recent years, a number of advances have been made in making use of the predictive value of the NLR. An increased NLR is strongly associated with disease severity and outcome. Pichler et al confirmed that increased NLR in patients with renal clear cell carcinoma is an independent risk factor for overall survival, which may reflect a higher risk of severe disease.^[12]^ NLR is an important inflammatory parameter. Inflammation is defined as 1 of the 10 characteristics associated with tumors. Tumer^[13]^ used HE staining to identify inflammatory cells that had infiltrated tumor tissue. Statistical analysis showed that the number of inflammatory cells infiltrating a tumor was positively correlated with overall survival and relapse-free survival of cancer patients, while the systemic inflammatory response was negatively correlated with the prognosis of cancer patients. Patients with low inflammatory cell infiltration and high peripheral blood NLR had a significantly worse prognosis than those with high inflammatory cell infiltration and low peripheral blood NLR. The combination of histological examination and routine blood data is more beneficial for determining cancer prognosis. Metastatic cancer is a reflection of advanced disease, and Nakayama et al^[14]^ considered that peripheral blood NLR can be used as an independent determinant of cancer metastasis. Cho et al^[15]^ found that peripheral blood NLR prior to treatment played a guiding role in the evaluation of chemotherapy efficacy and prognosis of survival time for metastatic advanced cancer.

The Pearson chi-square, Spearman correlation analysis, univariate and multivariate Cox regression analysis, and receiver operating characteristic (ROC) curve were used in this study to explore and verify the relationship between NLR level and overall survival of patients with MccRCC. This may provide new directions for treatment and prediction of survival in patients with MccRCC.

## 2 Methods

### 2.1 Patients and groups

The study participants were 100 patients who were diagnosed with MccRCC using MSCT and who took part in a prospective study at Urumqi Friendship Hospital, China, between January 2012 and January 2020. The patients were divided into two groups according to overall survival based on their average survival time: short survival-time group (59 cases) and long survival-time group (41 cases). Patients’ clinical data were then analyzed to explore the role of NLR in MccRCC. The start point was when a participant was diagnosed with MccRCC by multi-slice spiral CT. The end point was when a participant died from MccRCC.

### 2.2 Ethics and patient consent

This study was approved by the Ethics Committee of the Urumqi Friendship Hospital. Written informed consent was obtained from all patients.

### 2.3 Inclusion and exclusion criteria

The inclusion criteria were 18 to 85 years old, diagnosed with MccRCC using MSCT, patients without surgical history, and good cooperation of patients and their families.

Exclusion criteria included age <18 years or >85 years; patients with poor cardiac, pulmonary, liver, or kidney function; and patients requiring emergency surgery.

### 2.4 Collection of clinical indicators

Age, sex, neutrophil count, lymphocyte count, blood glucose, NLR, blood urea nitrogen, and serum creatinine of patients with MccRCC were carefully recorded. Furthermore, we divided each indicator into two groups based on the average value. For all patients, NLRs were determined upon diagnosis and prior to any treatment. Short survival time was defined when the patients’ survival time was ≤30 months, and long survival time was defined when the patients’ survival time was >30 months. A neutrophil count <4.05 × 10^9^ was defined as low, and a neutrophil count >4.05 × 10^9^ was defined as high. A lymphocyte count <2.10 × 10^9^ was defined as low, and a lymphocyte count >2.10 × 10^9^ was defined as high. Blood glucose <5 mmol/L was defined as low, and blood glucose >5 mmol/L was defined as high. NLR <1.16 was defined as low, and NLR >1.16 was defined as high. Blood urea nitrogen <5.11 mmol/L was defined as low, and blood urea nitrogen >5.11 mmol/L was defined as high. Serum creatinine <75 μmol/L was defined as low, and serum creatinine >75 μmol/L was defined as high.

### 2.5 Statistics

The data were expressed as numbers and percentages. Associations between the clinical parameters and survival time of patients with MccRCC were analyzed using Pearson's chi-squared test. The Spearman-rho test was executed to compare clinical data and overall survival (OS) for the correlation analysis. Univariate and multivariate Cox regression analysis was used to calculate the hazard ratio (HR) of survival time for potentially correlated factors. We also used the Kaplan–Meier method to explore OS. The area under the curve (AUC) of the NLR was compared using a ROC curve to analyze the relationship between NLR and MccRCC. All statistical analyses were conducted using SPSS software, version 21.0 (IBM Corp, Armonk, NY). A *P*-value <0.05 was considered statistically significant.

## 3 Results

### 3.1 Associations between patient characteristics and OS of MccRCC based on the χ^2^ test

Table 1 summarizes the possible relationship between a patient's clinical factors and overall survival, according to Pearson's chi-square test. Among individuals, the NLR was significantly correlated with overall survival (*P* < .001). However, there were no significant correlations between survival time and sex (*P* = .694), age (*P* = .508), neutrophil count (*P* = .280), lymphocyte count (*P* = .147), blood glucose (*P* = .091), blood urea nitrogen (*P* = .345), or serum creatinine (*P* = .494) in patients with MccRCC (Table 1).

**Table 1:** Relevant characteristics of patients with MccRCC and overall survival.

		Overall survival		
Parameters		Short (%)	Long (%)	*P*
Sex				.694
Male	56	34 (34.0%)	22 (22.0%)	
Female	44	25 (25.0%)	19 (19.0%)	
Age				
≤65	38	24 (24.0%)	14 (14.0%)	.508
>65	62	35 (35.0%)	27 (27.0%)	
Neutrophil count				
Low	43	28 (28.0%)	15 (15.0%)	.280
High	57	31 (31.0%)	26 (26.0%)	
Lymphocyte count				
Low	55	36 (36.0%)	19 (19.0%)	.147
High	45	23 (23.0%)	22 (22.0%)	
Blood glucose				
Low	54	36 (36.0%)	18 (18.0%)	.091
High	46	23 (23.0%)	23 (23.0%)	
NLR				
Low	46	5 (5.0%)	41 (41.0%)	<.001^∗^
High	54	54 (54.0%)	0 (0.0%)	
Blood urea nitrogen				
Low	52	33 (33.0%)	19 (19.0%)	.345
High	48	26 (26.0%)	22 (22.0%)	
Serum creatinine				
Low	48	30 (30.0%)	18 (18.0%)	.494
High	52	29 (29.0%)	23 (23.0%)	

Pearson's chi-squared test was used.

∗ *P* < .05.

### 3.2 Further associations between patients’ characteristics and OS in MccRCC cases using Spearman's correlation test

To determine whether underlying correlational factors in patients with MccRCC have a significant impact on OS, a further correlation analysis was performed. Spearman's correlation coefficient showed a significant correlation between OS and NLR (ρ = −0.903, *P* < .001). However, there were no further associations between survival time of patients with MccRCC and sex (ρ = −0.039, *P* = .698), age (ρ = 0.066, *P* = .513), neutrophil count (ρ = 0.108, *P* = .285), lymphocyte count (ρ = 0.145, *P* = .150), blood glucose (ρ = −0.169, *P* = .093), blood urea nitrogen (ρ = 0.094, *P* = .350), or serum creatinine (ρ = 0.068, *P* = .499) (Table 2).

**Table 2:** The relationship between characteristics of patients and overall survival.

	Overall survival	
Characteristics	ρ	*P*
Sex	0.039	.698
Age	0.066	.513
Neutrophil count	0.108	.285
Lymphocyte count	0.145	.150
Blood glucose	0.169	.093
NLR^∗^	–0.903	<.001^∗^
Blood urea nitrogen	0.094	.350
Serum creatinine	0.068	.499

Spearman correlation test was used.

∗ *P* < .05.

### 3.3 Univariate Cox regression analysis for the proportional hazards analysis of correlative factors related to OS in patients with MccRCC

**Figure 1:**
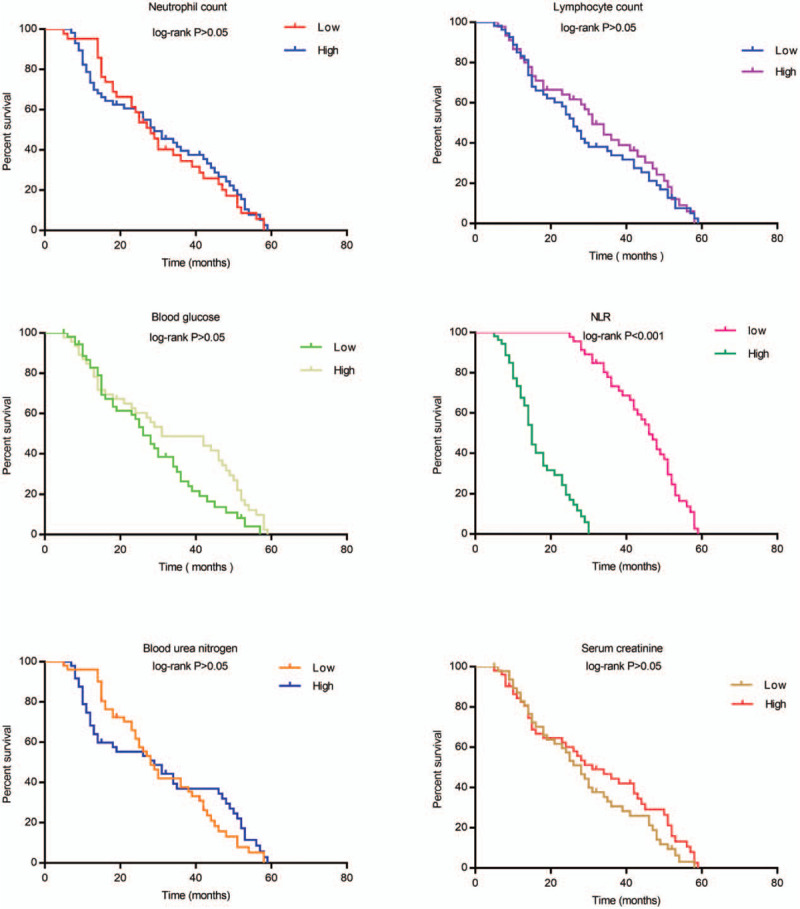
A comparison of various clinical parameters and their effect on overall survival in patients with MccRCC.

Table 3 describes the HRs and 95% confidence intervals (95% CI) between clinically relevant factors and overall survival time in patients with MccRCC. For the factor of NLR, OS in the high-level NLR group was significantly lower than in the low-level NLR group, and the HR was 32.467 (95%CI, 12.260–85.979, *P* < .001). However, there were no significant differences in OS in cases of MccRCC related to any of the following factors: sex (HR = 0.725, 95%CI, 0.048–1.123, *P* = .150), age (HR = 0.833, 95%CI, 0.537–0.292, *P* = .414), neutrophil count (HR = 0.949, 95%CI, 0.620–1.453, *P* = .811), lymphocyte count (HR = 0.879, 95%CI, 0.575–1.342, *P* = .549), blood glucose (HR = 0.642, 95% CI, 0.411–1.002, *P* = .051), blood urea nitrogen (HR = 0.919, 95%CI, 0.598–1.413, *P* = .702), and serum creatinine (HR = 0.768, 95%CI, 0.501–1.175, *P* = .223) (Fig. 1, Table 3).

**Table 3:** Characteristics and their effect on OS based on univariate Cox proportional regression analysis.

		OS		
Characteristics		HR	95% CI	*P*
Sex		1		.150
Male	56			
Female	44	0.725	0.048–1.123	
Age				
<65 years	38	1		.414
≥65 years	62	0.833	0.537–0.292	
Neutrophil count				
Low	43	1		.811
High	57	0.949	0.620–1.453	
Lymphocyte count				
Low	55	1		.549
High	45	0.879	0.575–1.342	
Blood glucose				
Low	54	1		.051
High	46	0.642	0.411–1.002	
NLR^∗^				
Low	46	1		<.001^∗^
High	54	32.467	12.260–85.979	
Blood urea nitrogen				
Low	52	1		.702
High	48	0.919	0.598–1.413	
Serum creatinine				
Low	48	1		.223
High	52	0.768	0.501–1.175	

95% CI = 95% confidence interval; HR = hazard ratio; OS = overall survival.

∗ *P* < .05.

### 3.4 Analysis of OS based on multivariate Cox regression analysis for the proportional hazards of related characteristics

To effectively control the influence of confounding factors, all factors were simultaneously incorporated into the multivariate Cox regression model. Table 4 shows the results of the multivariate Cox proportional regression analysis. NLR (HR = 50.676, 95% CI, 17.543–146.390, *P* < .001) and neutrophil count (HR = 1.874, 95% CI, 1.146–3.063, *P* = .012) were significantly associated with OS, whereas sex (HR = 0.668, 95%CI, 0.416–1.070, *P* = .093), age (HR = 0.847, 95%CI, 0.531–1.351, *P* = .485), lymphocyte count (HR = 1.399, 95%CI, 0.886–2.207, *P* = .149), blood glucose (HR = 0.982, 95%CI, 0.592–1.629, *P* = .945), blood urea nitrogen (HR = 1.359, 95%CI, 0.531–1.351, *P* = .485), and serum creatinine (HR = 0.914, 95%CI, 0.586–1.426, *P* = .692) showed no significant correlation with OS (Table 4).

**Table 4:** Characteristics and their effect on OS based on multivariate Cox regression analysis.

	OS		
Characteristics	HR	95% CI	*P*
Sex	0.668	0.416–1.070	.093
Age	0.847	0.531–1.351	.485
Neutrophil count^∗^	1.874	1.146–3.063	.012^∗^
Lymphocyte count	1.399	0.886–2.207	.149
Blood glucose	0.982	0.592–1.629	.945
NLR^∗^	50.676	17.543–146.390	<.001^∗^
Blood urea nitrogen	1.359	0.834–2.215	.218
Serum creatinine	0.914	0.586–1.426	.692

95% CI = 95% confidence interval; HR = hazard ratio; OS = overall survival.

∗ *P* < .05.

### 3.5 The ROC curve was used to analyze related factors in patients with MccRCC

**Figure 2:**
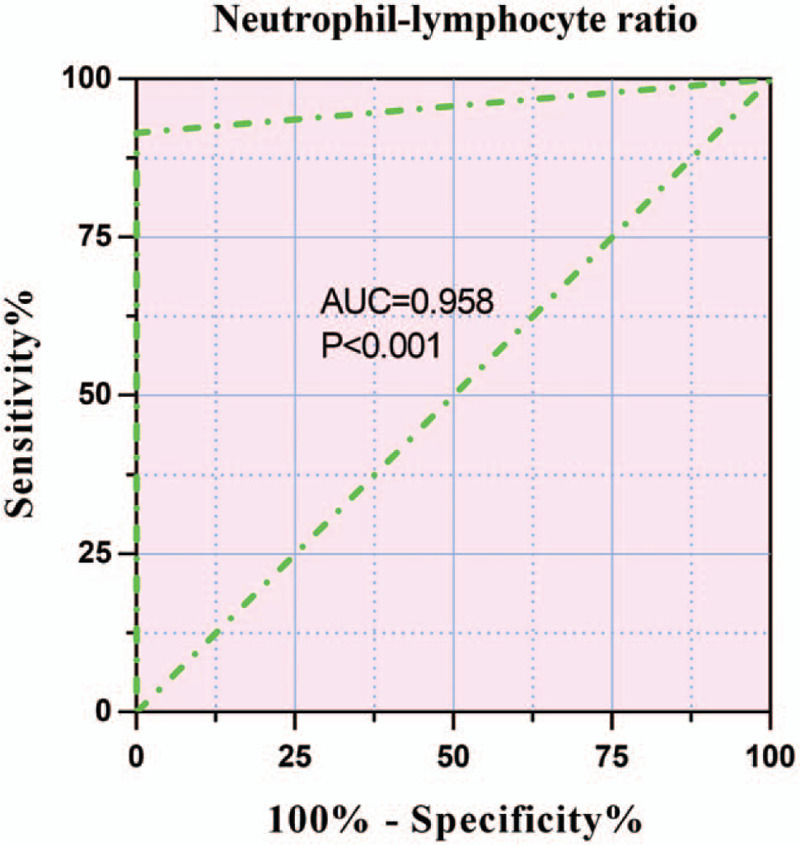
The ROC curve of NLR for cases of MccRCC.

The ROC curve analysis showed that the AUC of the NLR for predicting OS of patients with MccRCC was 0.958 (95%CI, 0.915–1.000, *P* < .001) (Fig. 2). However, sex (AUC = 0.520, 95%CI, 0.404–0.635, *P* = .737), age (AUC = 0.533, 95%CI, 0.418–0.648, *P* = .580), neutrophil count (AUC = 0.554, 95%CI, 0.440–0.669, *P* = .357), lymphocyte count (AUC = 0.573, 95%CI, 0.459–0.688, *P* = .214), blood glucose (AUC = 0.586, 95%CI, 0.471–0.700, *P* = .147), blood urea nitrogen (AUC = 0.548, 95%CI, 0.433–0.663, *P* = .416), and serum creatinine (AUC = 0.535, 95%CI, 0.419–0.650, *P* = .556) were not good predictors of OS in patients with MccRCC (Table 5).

**Table 5:** Receiver operator characteristic curve analysis of N/L for MccRCC.

	MccRCC		
Characteristics	AUC	*P*	95%CI
Sex	0.520	.737	0.404–0.635
Age	0.533	.580	0.418–0.648
Neutrophil count	0.554	.357	0.440–0.669
Lymphocyte count	0.573	.214	0.459–0.688
Blood glucose	0.586	.147	0.471–0.700
NLR	0.958 ^max^	<.001^∗^	0.915–1.000
Blood urea nitrogen	0.548	.416	0.433–0.663
Serum creatinine	0.535	.556	0.419–0.650

AUC = area under curve; ^max^ = the maximum of AUC; NLR = neutrophil–lymphocyte ratio; MccRCC = minute clear cell renal cell carcinoma.

∗ *P* ≤ .05.

### 3.6 Analysis of metastasis-free survival and cancer-specific survival

**Figure 3:**
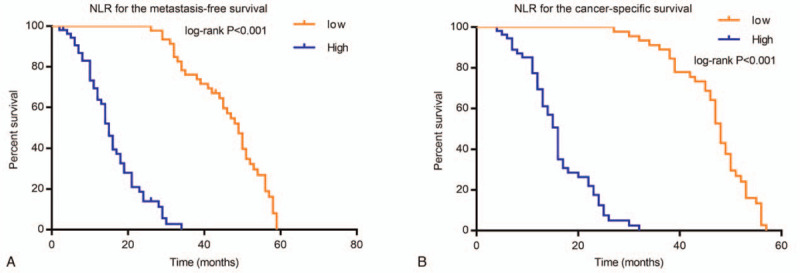
Analysis of metastasis-free survival and cancer-specific survival. (A) Role of the NLR for the metastasis-free survival. (B) Role of the NLR for cancer-specific survival.

Metastasis-free survival in the high-level NLR group was significantly lower than metastasis-free survival in the low-level NLR group; the HR was 21.51 (95%CI, 11.50–40.22, *P* < .001). Furthermore, cancer-specific survival in the high-level NLR group was significantly lower than in the low-level NLR group; the HR was 26.56 (95%CI, 13.99–50.39, *P* < .001) (Fig. 3).

## 4 Discussion

In this study, Pearson's chi-square test, Spearman correlation analysis, univariate and multivariate Cox regression analysis, and ROC curve analysis were used and determined that NLR is closely related to MccRCC outcomes. The higher their NLR, the worse the prognosis for patients with MccRCC.

Kidney cancer, which accounts for 2% to 3% of all cancers and is on the rise, is a highly malignant tumor of the urinary system.^[16,17]^ In recent years, the widespread use of multi-slice CT (MSCT) and other radiographic imaging techniques has led to a significant increase in the percentage of small renal tumors that are accidentally detected during the asymptomatic stage. MccRCC has the characteristics of being clinically asymptomatic, with small lesions that are easy to miss, but MSCT offers a means to avoid missing small lesions and improve the detection and diagnosis of renal microtumors; therefore, it is widely recommended for the diagnosis of MccRCC.^[18]^

In contrast, laboratory tests, including C-reactive protein (CRP), urea nitrogen, creatinine, liver function, complete blood count (CBC), hemoglobin, blood glucose, alkaline phosphatase, and lactate dehydrogenase, play an important role in the diagnosis of minimal renal carcinoma. Among these, CRP, which is a serum marker of systemic inflammation, showed promise as a prognostic tool in patients with renal cell carcinoma.^[19]^ Pilskog et al confirmed that baseline CRP may be a useful biomarker in mRCC treatment plans.^[20]^ Kalogirou et al demonstrated the effectiveness of determining preoperative CRP level as a prognostic marker of survival in a cohort of patients with mRCC undergoing cell-reducing nephrectomy (CN).^[21]^

The NLR is a simple, cost-effective and readily available biomarker, which is commonly seen in various malignant tumors, inflammatory states, and inflammatory diseases.^[11,22]^ An increase in NLR is the result of both increased circulating neutrophils and decreased lymphocytes that cause systemic inflammation. NLR has been reported to be closely related to serum CRP levels. Meanwhile, NLR has also shown efficacy as an alternative marker for systemic inflammation in critically ill patients, malignancies, and chronic diseases such as end-stage kidney disease and diabetes.^[23,24]^ Eochagain et al demonstrated that inflammation and immunosuppression are involved in the pathogenesis of cancer and that an increased NLR reflects these processes and is associated with adverse outcomes of cancer.^[25]^ Selahattin et al found that NLR can be used as an indicator for preoperative diagnosis of renal cell carcinoma.^[26]^ Studies by Palin et al have found that NLR is significantly associated with increased colorectal cancer mortality. NLR is an inexpensive, simple, and effective clinical tool for predicting the prognosis of colorectal cancer patients.^[27]^ Mellor et al have shown that NLR is an important prognostic indicator of OS and DFS after R0 resection for gastric cancer, but its critical value remains unclear.^[28]^ Pichler^[12]^ found that in a large, validated European study of NLR pretreatment prognosis in 678 patients with renal cell carcinoma that preoperative NLR elevation was associated with poor OS, but not with cancer-specific outcomes. Ersan's^[29]^ research showed that there was a linear correlation between NLR and tumor size in renal cell carcinoma. Therefore, NLR is a cheap to measure biomarker that could be used to predict tumor size, and thus it may be used to gain insights into the prognosis of patients with RCC. Vincenzo's^[30]^ study concluded that higher NLR resulted in worse OS and progression-free survival (PFS) in the overall population (OS pooled HR 1.80; 95%CI: 1.61–2.00; *I*^2^ 45%; PFS pooled HR of 1.69; 95%CI: 1.42–2.01; *I*^2^ 81%). In addition, NLR is an easily accessible biomarker that can be used determine a prognosis in renal cell carcinoma. Selahattin^[26]^ found that the NLR may be a useful diagnostic biomarker parameter for renal cell carcinoma in the preoperative period. The findings of this earlier research are in agreement with those of our study. What was innovative about our study was that we targeted MccRCC diagnosed using multi-slice spiral CT; there has been little similar research into MccRCC.

There were some limitations to our study. First, due to the limited number of patients in our hospital, elderly people (aged >85 years) were not included in this study. Second, this research was a single-center study, with no additional centers included for a large-scale investigation. In the future, we would make efforts to address these aspects.

## 5 Conclusion

This study confirmed that the NLR plays a crucial role in the prognosis of patients with MccRCC. The higher the NLR level, the lower the survival time of patients with MccRCC. This finding may provide a new perspective for the treatment and prognosis of patients with MccRCC.

## Author contributions

**Conceptualization:** Lingjun Qi.

**Data curation:** Xiaoxin Chai.

**Formal analysis:** Li Chen.

**Funding acquisition:** Lingjun Qi.

**Investigation:** Qian Ma.

**Methodology:** Li Chen, Lingjun Qi.

**Project administration:** Qian Ma, Xiaoxin Chai.

**Resources:** Jing Zhang.

**Software:** Jing Zhang.

**Supervision:** Xiaoxin Chai.

**Validation:** Jing Zhang.

**Visualization:** Qian Ma.

**Writing – original draft:** Li Chen.

**Writing – review & editing:** Xiaoxin Chai.
